# Can the establishment of state-level urban agglomeration stimulate enterprise innovation?—Taking Yangtze River Delta and Pearl River Delta as an example

**DOI:** 10.1371/journal.pone.0273154

**Published:** 2022-08-25

**Authors:** Kai Zhao, Huahua Huang, Wanshu Wu

**Affiliations:** 1 School of Economics, Qingdao University, Qingdao, China; 2 School of Statistics, Huaqiao University, Xiamen, China; 3 College of Architecture and Urban Planning, Qingdao University of Technology, Qingdao, China; Northeastern University (Shenyang China), CHINA

## Abstract

This study uses a quasi-experimental method, Geographic Regression Discontinuity Design (GRDD), to evaluate the actual effect of establishing Yangtze River Delta and Pearl River Delta urban agglomerations on enterprise innovation. GRDD is a design in which a geographic boundary splits the units into treated and control areas in an as-if random fashion, and the shortest distances from each enterprise’s location to the boundary of urban agglomeration calculated by ArcGIS are considered as the running variable. The actual effect can be identified by the probability of receiving treatment jumps discontinuously at the known cutoff. It is shown that the establishment of Yangtze River Delta and Pearl River Delta urban agglomerations can significantly improve the enterprise innovation, and this outcome is verified by rigorous robustness tests including the placebo test with pseudo-boundary, the bandwidth sensitivity test, the parametric test with different functional forms and the extreme value test. Further, the influence mechanisms of state-level urban agglomerations promoting enterprise innovation are explored by Staggered DID. It is confirmed that the urban agglomeration construction can promote enterprise innovation through financial support and regional coordination channels.

## 1 Introduction

Economic globalization needs strengthen the development of urban agglomeration, which has become the most important modern economic development mode. The top 40% of the world’s urban agglomerations contribute 66% of the global economy and 85% of scientific and technological innovations [1]. “First experimenting and then spreading” is the consistent thinking of China’s central and local governments to promote various reforms. As a creative achievement to promote China’s economic development and accelerate the urbanization process, the exploration and practice of urban agglomeration construction is surpassing the national and administrative boundaries and becoming a “node” connecting the global economy, which is related to the realization of the 14th “Five-Year Plan” and the “Long-term Goal of 2035” [2].

Under the background of economic globalization and regional integration, the importance of innovation is self-evident. No matter a country, a region or even an enterprise, the economic competitive advantage comes from the constantly improving innovation ability. Although some new global problems (*i*.*e*. epidemic of COVID-19, Sino-US trade friction) have changed the external environment in recent years, innovation is still the key to gain competitive advantage and promote the high-quality development. Enterprises, as the main body of innovation, play an important role in promoting the high-quality development of China, while state-level urban agglomerations, as the key carrier of innovation-driven, provide a novel perspective for understanding local government behavior, enterprise innovation strategies and China’s economic growth. Building a scientific and rigorous analysis framework to clarify the relationship between China’s urban agglomeration construction and enterprise innovation can expand the evaluation perspective of China’s urban agglomeration policy, and provide useful ideas for realizing high-quality economic development by encouraging enterprise innovation [3].

Yangtze River Delta (henceforth, YRD) is the most comprehensive state-level urban agglomeration in China, located in the alluvial plain at the mouth of the Yangtze River. The YRD urban agglomeration covers 26 cities, including Shanghai, Nanjing and Hangzhou, with a land area of 211,700 square kilometers and a total population of 150 million (in 2020), accounting for one-fifth of China’s GDP. Pearl River Delta (henceforth, PRD) is the state-level urban agglomeration with the strongest economic vitality in China. It is composed of 9 cities including Guangzhou, Shenzhen and Foshan, with a total area of 56,000 square kilometers and a total population of 70 million people (in 2020). Both YRD and PRD urban agglomerations are bases for scientific and technological innovation, important platforms for China to participate in economic globalization, strategic areas for China’s modernization. They are the two most advantageous urban agglomerations in China in terms of location and policy. On the whole, the YRD and PRD have become China’s urban agglomerations with the largest population inflow and the highest innovation output. Therefore, clarifying the actual effect of establishment of YRD and PRD urban agglomerations on enterprise innovation has important values for optimizing government system and encouraging enterprise innovation. Specifically, as far as the government is concerned, the accurate evaluation of the impact of urban agglomeration establishment on enterprise innovation and the identification of the preconditions and conditions for the policy implementation can provide important reference for the central and local governments to formulate effective development strategies, and provide a reliable basis for the introduction of future policies. As far as enterprises are concerned, discussing the relationship between the construction of YRD and PRD urban agglomerations and the innovation of enterprises is helpful to make innovation investment decisions at the micro-level and provide certain reference and theoretical guidance for enterprises to optimize the investment structure.

Selection and endogeneity are often key threats to inference in the social science. Recently, analysts have turned to natural experiments and quasi-experimental methods as one way to overcome these obstacles in observational studies [4]. Although the Difference-In-Difference (henceforth, DID) method can effectively identify the impact of the establishment of YRD and PRD urban agglomerations, there is still a lack of analysis on the spatial role of urban agglomeration. To this end, we use a quasi-experimental method, Geographic Regression Discontinuity Design (henceforth, GRDD), to evaluate the actual effect of establishing YRD and PRD urban agglomerations on enterprise innovation from the spatial viewpoint.

The contributions of this study are mainly reflected in the following two aspects. First, the establishment of urban agglomeration is a strategic plan put forward by the state according to the current economic development situation, and it is the comprehensive product of regional economic situation and administrative planning. Investigating the impact of state-level urban agglomeration establishment across administrative divisions on micro enterprise innovation, provides a beneficial supplement for China’s location-oriented policy evaluation. Second, examining the actual effect of urban agglomeration establishment by GRDD, can effectively take the impact of “spatial layout” and “policy events” into account. While overcoming the possible interference of endogenous problems on the empirical results, this study provides a solid micro-foundation for how to practice and promote “innovation-driven” and “high-quality development” in the new era.

The paper is organized as follows. In the next section, we provide a brief literature review. In section 3, we present the research design, identification strategy and estimation method. In section 4, the data source and the preliminary exploration are discussed. In section 5, we discuss the estimated results, and verify the reliability of the results. In section 6, we draw the main conclusions and policy recommendations.

## 2 Literature review

### 2.1 Urban agglomeration

There has been a long debate about the role of urban agglomeration in the literature on why some regions successfully achieve growth, while other regions stagnate or decline [5]. Since Gottmann puts forward the concept of urban agglomeration (Megalopolis) [6], a large number of scholars begin to explore urban agglomeration from the perspectives of geography, regional economics, urban economics, environmental science and other disciplines [7]. These studies not only reflect the characteristics and the evolution of urban agglomerations, but also include the prediction and optimization suggestions for the future development, providing theoretical basis for the strategic planning of world urban agglomerations. The literature on urban agglomeration can be roughly divided into three stages: the initial stage (2000–2007), the developing stage (2008–2014), and the mature stage (2015–2022).

At the initial stage, scholars focus on the agglomeration form of “city-industry”, and try to explore the geographical scope of external economic operation [8]. The related research in this stage is mainly to investigate the formation process, population flow patterns and industrial cluster types of urban agglomerations in the northeastern United States and Europe based on qualitative methods and case studies [9, 10]. At the developing stage, scholars pay attention to the development of urban agglomeration caused by urban network and urban cooperation. It is found that urban expansions not only lead to regional fragmentation, but also generate new regional associations, and the huge spatial scale will help to form new regional networks and spatial associations across metropolitan areas [11]. The study of urban agglomeration in this stage changes from qualitative to quantitative models, and the cellular automata simulation, the multiple linear regression and the gravity model become analytical tools to study the evolution of urban agglomeration [12–14]. In addition, the night-light data and the traffic data are more and more widely used in urban agglomeration study [15, 16]. With the development of urbanization and urban agglomeration, the evaluation and the mechanism analysis of sustainable development of economy and environment become the center of attention, and the research on urban agglomeration enters a mature stage. Many empirical studies evaluate the development benefits of urban agglomerations from the perspective of economic efficiency. These studies mainly include: building the empirical framework of spatial structure affecting the economic efficiency of urban agglomeration [17]; discussing the spatial scale conditions of urban agglomeration scale benefit [18]; evaluating the policy performance of establishing urban agglomeration [7]; exploring the relationship between urban agglomeration spatial integration and industrial coordinated development [19]. Besides, the development of urban agglomeration inevitably involves resource consumption [20] and environmental pollution [21].

The research on China’s urban agglomeration mainly involves four aspects: first, the horizontal comparison of the development of urban agglomerations and longitudinal spatiotemporal evolution analysis [22]; second, the scheme optimization and adjustment of urban agglomeration construction [23]; third, the impact of urban agglomeration on economic development, culture, ecological environment and ecological efficiency [24, 25]; fourth, the spatial role of urban agglomerations [26, 27]. Scholars mainly investigate the effects of China’s urban agglomeration from macro and meso perspectives. Firstly, from the macro perspective, the actual impact of urban agglomeration on high-quality economic development [28] and the promoting effect of urban agglomeration construction on China’s regional development [29] are investigated by taking the Yangtze River Delta, Beijing-Tianjin-Hebei and other urban agglomerations in China as examples. Secondly, from the meso perspective, the promoting effects of Yangtze River Delta urban agglomeration on regional labor productivity [30], total factor productivity, efficiency change and technology change [31] are discussed in-depth.

### 2.2 Enterprise innovation

Generally, enterprise innovation is affected by external environmental factors and internal control factors. The external environmental factors mainly involve the horizontal competition environment [32], market operation environment [33], thermal comfort environment [34], and policy implementation environment [35] faced by enterprises in the process of innovation. The horizontal competition environment can significantly adjust the performance and innovation spirit of enterprises. Under the precondition that the performance is fixed, the fiercer the competition in the same industry, the lower the willingness of enterprises to innovate [36]. The marketization degree is one of the important indexes to evaluate the market operation environment, and the difference of marketization degree in different regions is often an important reason for the heterogeneity of innovation behaviors [37].

The internal control factors that affect enterprise innovation mainly involve ownership and management [38, 39], and the impact of internal control factors on enterprise innovation is still inconclusive. First, from the perspective of ownership, Chen et al. confirms that the stronger the control over enterprises, the more beneficial it is to encourage enterprises to increase innovation input and improve innovation output [40]. However, some studies find that there is an “inverted U-shaped” relationship between the level of ownership and the enterprise innovation, and too high or too low ownership will reduce the willingness of enterprises to invest in R&D [41], and may hinder the innovation output [42]. Second, from the perspective of management, moderate concentration of management can ensure the efficient execution of enterprises and provide a strong guarantee for enterprises to carry out innovation activities [43]; excessively centralized management may lead to inefficient decision-making quality and further generate a negative impact on enterprise innovation [44]; excessively decentralized management may lead to decision-making conflicts, which in turn inhibits the innovation of enterprises [45].

### 2.3 Policy evaluation and GRDD

Knowledge resource related to innovation activities play an important role in high-quality economic development. As the government’s knowledge resource investment has been rising, the importance of policy evaluation has become pronounced [46, 47]. Regression Discontinuity Design (henceforth, RDD), as a mainstream quasi-experimental approach, has been gradually applied to economics since the late 1990s [48]. Lee and Lemieux summarize the promise that surrounds this design, attributing the recent wave of RDD studies to “the belief that the RDD is not ‘just another’ evaluation strategy and that causal inferences from RDD are potentially more credible than those from typical ‘natural experiment’ strategies” [49]. Wang et al. use the RDD to empirically test the influence of the establishment of the Shanghai pilot free-trade zone on the green total factor productivity [50]. Moreover, analysts use RDD to recover experimental benchmarks, which have only bolstered their credibility [51]. By taking the boundaries of regions or regional policies as running variables, RDD can judge individuals in the treatment group or control group by their relative geographical boundary positions, thus constituting the Geographic RDD (GRDD).

One of the earliest and most famous examples of exploiting geographic variation to estimate causal effects is the study by Card and Krueger, who estimate the effect of increasing the minimum wage on employment by comparing fast-food restaurants in New Jersey (where the minimum wage is increased) to restaurants in adjacent eastern Pennsylvania [52]. In political science, political boundaries are often associated with variation in key treatments such as national or state institutions. For example, Posner uses the colonial border between Zambia and Malawi, which is drawn by the British South African Company and split two different ethnic groups, to study the political salience of cultural cleavages [53]. GRDD are an increasingly popular type of natural experiment in political science, and have been recently used to study a variety of topics [54]. Dell is the first to take geographical distance as a running variable, and uses GRDD to explore the impact of the labor service system implemented by the Spanish colonial government in Peru on the local economic development [55]. Subsequently, scholars begin to use GRDD to study Chinese problems. Chen et al use the “Qinling Mountains and Huaihe River” central heating boundary as the geographical cutoff line to study the impact of central heating on air quality, and it is found that coal burning for heating in the north of “Qinling Mountains and Huaihe River” line reduces average life expectancy [56]. Xin and Xu start with a border of the puppet Manchukuo, and deeply discusses the long-term impact of the puppet Manchukuo colonial rule on the regional economy of their jurisdiction [57]. Yu and Wang take the lead in applying GRDD to urban agglomeration performance evaluation [58]. Due to the large expansion scope and strong observability of the YRD urban agglomeration, Deng and Li take the capacity expansion of the YRD urban agglomeration as a quasi-natural experiment by means of GRDD [2]. The actual impact of urban agglomeration expansion in the YRD is identified by comparing the performance changes of 60 cities near the expansion boundary in 2010.

To sum up, there are still the following problems to be improved. First, there are still differences on the delineation of urban agglomeration, and few literatures discuss the micro-enterprise innovation within the scope of urban agglomeration. Second, existing studies mostly use OLS or DID methods to investigate the impact of location-oriented policies such as urban agglomeration, but there is still a lack of analysis on the spatial role of urban agglomeration.

## 3 Research design

### 3.1 Setup and notation

GRDD is a design in which a geographic or administrative boundary splits the units into treated and control areas in an as-if random fashion. It is a special case of the RDD with two arbitrary scores (coordinate systems like latitude and longitude). We compare the enterprises in a treated area (YRD and PRD urban agglomerations) to enterprises in a control area, which we denote by A^T^ and A^C^, respectively. We adopt the potential outcomes framework and assume that enterprise *i* has two potential outcomes, *Y*_*i*1_ and *Y*_*i*0_, which correspond to levels of treatment *T*_*i*_ = 1 and *T*_*i*_ = 0, respectively. In this context, *T*_*i*_ = 1 denotes that enterprise *i* is within YRD and PRD urban agglomerations (A^T^) and *T*_*i*_ = 0 denotes that enterprise *i* is out of urban agglomeration (A^C^). The observed outcome is *Y*_*i*_ = *T*_*i*_*Y*_*i*1_+(1−*T*_*i*_)*Y*_*i*0_, and the fundamental problem of causal inference is that we cannot observe both *Y*_*i*1_ and *Y*_*i*0_ simultaneously for any given enterprise, which implies that we cannot recover the individual effect *τ*_*i*_ = *Y*_*i*1_−*Y*_*i*0_.

We define the variable that uniquely represents enterprise *i*’s geographic location, and allows us to compute enterprise *i*’s distance to any point on the border. We use vectors, in bold, to simplify the notation. The geographic location of enterprise *i* is given by latitude and longitude, (*S*_*i*1_,*S*_*i*2_) = **S**_*i*_. We consider the set that collects the locations of boundary points **B** (cutoff), and denote a single point on the boundary by **b** = (*S*_1_,*S*_2_)∈B. Thus, A^T^ and A^C^ are the sets that collect, respectively, the locations that receive treatment and control. The treatment assignment is a deterministic function of **S**_*i*_, and can be written as *T*_*i*_ = *T*(**S**_*i*_), with *T*(**s**) = 1 for **s**∈A^T^ and with *T*(**s**) = 0 for **s**∈A^C^. This assignment has a discontinuity at the known boundary B, and the actual effect of the establishment of YRD and PRD urban agglomerations on the innovation activities of enterprises can be identified by comparing whether the innovation activities of enterprises on both sides of the border jump at the known boundary B.

### 3.2 Identification

GRDD is a particular case of the two-dimensional RDD, and geography creates a number of complications that are not necessarily common in nongeographic designs. Therefore, it is better to define the running variable (score) *S* as the shortest distance to the boundary. Enterprises that are close to the boundary in terms of this distance but on opposite sides of it are taken as valid counterfactuals for each other [59]. Specifically, enterprise *i* has distance *S*_*i*_ = *dist* if the distance from enterprise *i*’s location to the point on the boundary that is closest to *i* is equal to *dist*. If Pr(*T*_*i*_ = 1) = 1 for all *i* such that **s**_*i*_∈A^T^ and Pr(*T*_*i*_ = 0) = 1 for all *i* such that **s**_*i*_∈A^C^ (the discontinuity is sharp), then

$$\begin{array}{l} \tau (b)\equiv E\{Y_{i1}-Y_{i0}|S_{i}=b\}\\ \;=\underset{s^{T}\to b}{\lim}E\{Y_{i}|S_{i}=s^{T}\}-\underset{s^{C}\to b}{\lim}E\{Y_{i}|S_{i}=s^{C}\}\;for\;all\;b\in B \end{array}$$ (1)

In other words, we can identify one treatment effect *τ*(**b**) for every point **b** on the boundary, defining a treatment effect curve. The average parameter *τ* = *E*{*Y*_*i*1_−*Y*_*i*0_|**S**_*i*_∈**B**} could be obtained by integrating the *τ*(**b**) effects over the entire boundary. Following previous studies [60], we can write the average treatment effect *τ* as

$$\tau =\int_{s\in B}\tau (s)f(s|S\in B)ds=\frac{\int_{s\in B}\tau (s)f(s)ds}{\int_{s\in B}f(s)ds}$$ (2)

which can be easily recovered once the local effects *τ*(⋅) and the density *f*(⋅) are estimated at multiple boundary points.

### 3.3 Estimation

To estimate a conditional expectation of the outcomes as a function of the distance to the boundary, we follow the existing studies [4] and define *μ*(*x*) = *E*(*Y*|*X* = *x*) as the regression function of the observed outcome *Y* on some univariate *X*. Assume that the first *p*+1 derivatives of *μ*(*x*) at the point *X* = *x*_0_ exist, we can approximate *μ*(*x*) in a neighborhood of *x*_0_ by a Taylor expansion:

$$\mu (x)\approx \mu (x_{0})+\mu^{1}(x_{0})(x-x_{0})+\frac{\mu^{2}(x_{0})}{2}{\left(x-x_{0}\right)}^{2}+\ldots +\frac{\mu^{p}(x_{0})}{p!}{\left(x-x_{0}\right)}^{p}$$ (3)

where *μ*(*x*_0_),*μ*^1^(*x*_0_),…,*μ*^*p*^(*x*_0_) denote the first (*p*+1)^*th*^ derivatives of *μ*(*x*_0_). In local regression estimation, this polynomial is fitted locally, minimizing a weighted sum of squared residuals. The estimated coefficients $\hat{\beta }={\left({\hat{\beta }}_{1},{\hat{\beta }}_{2},\ldots ,{\hat{\beta }}_{p}\right)}^{\prime }$ are defined as

$$\hat{\beta }=\arg\;\underset{\beta }{\min}\sum_{i=1}^{N}{\left[Y_{i}-\sum_{j=0}^{p}\beta_{j}{\left(X_{i}-x_{0}\right)}^{j}\right]}^{2}w_{i}$$ (4)

with weight $w_{i}=\frac{1}{h}K(\frac{X_{i}-x_{0}}{h})$ for a given kernel function *K*(⋅) (for example, uniform kernel: $K(u)=\frac{1}{2}1\{|u|<1\}$; triangular kernel: K(u)=(1−|u|)1{|u|<1}) and bandwidth *h*. Using this local polynomial estimator, we borrow the basic estimation approach from RDD, which involves estimating the left and right limits of *μ*(*c*), denoted *μ*^*l*^(*c*) and *μ*^*r*^(*c*) respectively, with a local polynomial of degree one, where *c* is a known cutoff in the score. The estimation of *μ*^*l*^(*c*) uses only observations to the left of *c*, similarly, estimation of *μ*^*r*^(*c*) uses only observations to the right of the cutoff. For given weights *w*_*i*_ and a scalar score *S*_*i*_, this involves computing the weighted regression of the observed outcome *Y*_*i*_ on a constant and *S*_*i*_−*c*. The estimated effect is then

$$\hat{\tau }=\hat{\mu^{r}(c)}-\hat{\mu^{l}(c)}$$ (5)

For a given point **b** on the boundary, we calculate the Euclidean distance between the location **S**_*i*_ of enterprise *i* and **b**. For every enterprise *i* in the sample, this distance is defined as *f*_**b**_(**S**_*i*_). Consider

$$\begin{array}{l} \mu {\left(b\right)}^{C}\equiv \underset{s^{C}\to b}{\lim}E\left\{Y_{i0}|f_{b}(S_{i})=f_{b}(s^{C})\right\}\\ \mu {\left(b\right)}^{T}\equiv \underset{s^{T}\to b}{\lim}E\left\{Y_{i1}|f_{b}(S_{i})=f_{b}(s^{T})\right\} \end{array}$$ (6)

Local linear regression is used to estimate the above functions and reduce estimation bias [61], namely

$$\begin{array}{l} ({\hat{\alpha }}_{b}^{C},{\hat{\beta }}_{b}^{C})=\arg\;\underset{\alpha_{b}^{C},\beta_{b}^{C}}{\min}\sum_{i\in A^{C}}\left\{{Y_{i}-\alpha_{b}^{C}-\beta_{b}^{C}[f_{b}(S_{i})-f_{b}(b)]}^{2}\right\}w_{ib}\\ ({\hat{\alpha }}_{b}^{T},{\hat{\beta }}_{b}^{T})=\arg\;\underset{\alpha_{b}^{T},\beta_{b}^{T}}{\min}\sum_{i\in A^{T}}\left\{{Y_{i}-\alpha_{b}^{T}-\beta_{b}^{T}[f_{b}(S_{i})-f_{b}(b)]}^{2}\right\}w_{ib} \end{array}$$ (7)

where $w_{ib}=\frac{1}{h_{b}}K(\frac{f_{b}(S_{i})-f_{b}(b)}{h_{b}})$ are a set of spatial weights, *h*_**b**_ is the bandwidth at the boundary point **b**. Kernel function *K*(⋅) mainly includes triangular kernel, uniform kernel and epanechnikov kernel. Since triangular kernel is more suitable for boundary estimation in local linear regression [49], we use triangular kernel function to minimize the above equations, and test the robustness with other kernel functions.

## 4 Data and preliminary exploration

This study takes listed enterprises within 100 kilometers on both sides of the boundary of YRD and PRD urban agglomerations in China from 2007 to 2019 as research samples. There are two reasons for selecting enterprises within 100km inside and outside the boundary of urban agglomeration as research samples: first, 100km inside the boundary of the urban agglomeration can cover all listed enterprises within the urban agglomeration. If the distance is too small (for example, 50km), some enterprises within urban agglomeration will not be included in the analysis; second, when designing the geographic regression discontinuity, the distance between the two sides of the boundary should be symmetrical far as possible.

### 4.1 Data source and variable description

**Fig 1:**
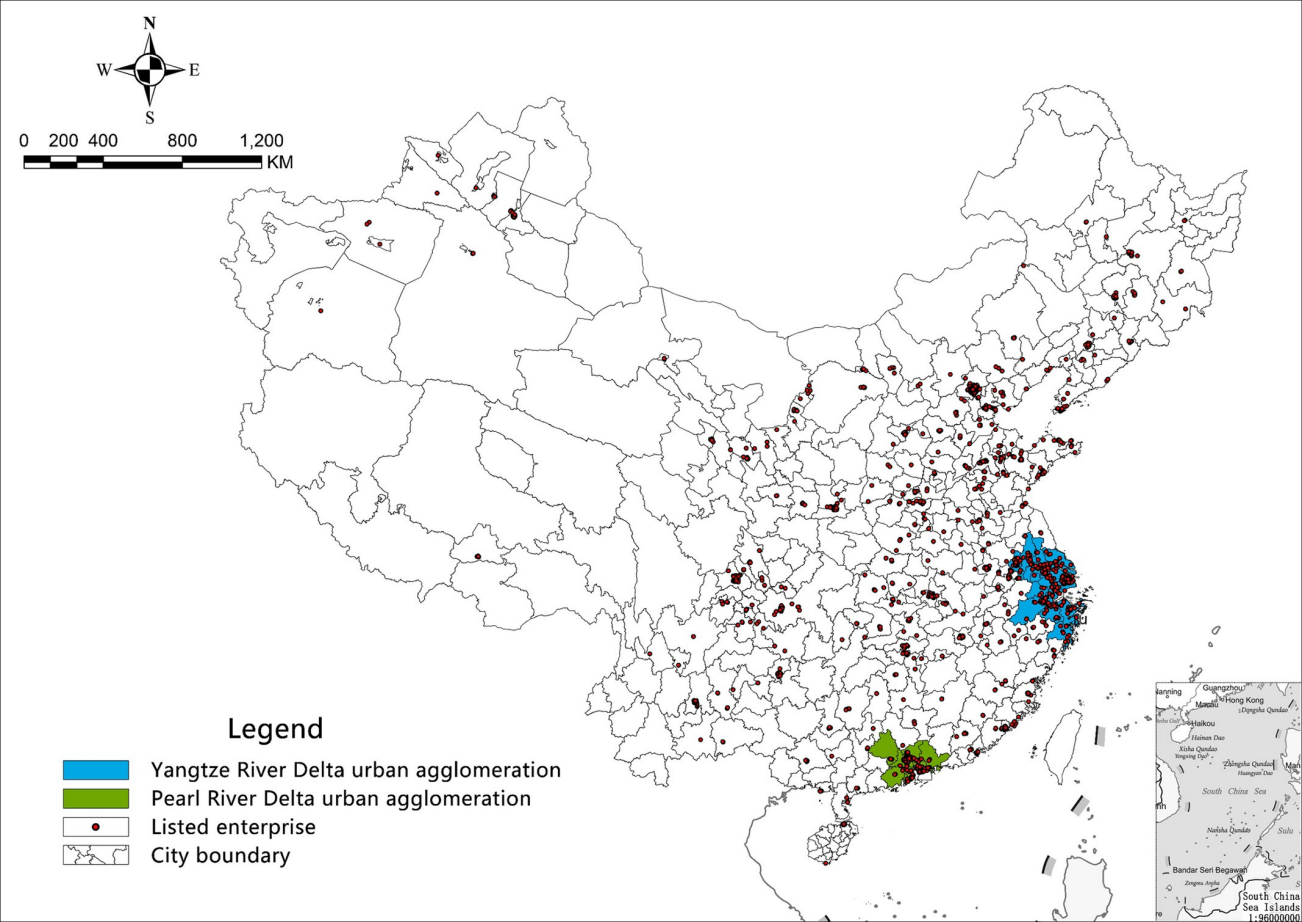
Urban agglomerations and listed enterprises.

The research data in this study consist of micro-enterprise data and urban-agglomeration-map data. Micro-enterprise data stem from the China Stock Market & Accounting Research (CSMAR) database (http://www.csmar.com/), mainly involving the China’s A-share listed enterprises from 2007 to 2019. Specifically, the patent data (such as patent applications, invention patent applications and patent grants) of enterprises comes from the “Patent Database of Listed Companies and Subsidiaries” in CSMAR database. Considering that the index of research and development investment in CSMAR database changed its statistical caliber in 2007, sample data after 2007 are selected for analysis to ensure the consistency of data statistical caliber. In addition, this study also processes the original data as follows: First, we eliminate ST, PT and samples with variable deletion. Secondly, in order to ensure the comparability of samples before and after the policy of YRD and PRD urban agglomerations, we eliminate enterprises that are listed after 2007 and delisted before 2019. Urban-agglomeration-map data stem from the “*Yangtze River Delta Urban Agglomeration Development Plan*” approved by the Chinese government in 2016 and the “*Pearl River Delta Urban Agglomeration Coordinated Development Plan (2004–2020)*” approved by the Chinese government in 2004. Fig 1 shows the location information of China’s listed enterprises and the boundary information about YRD and PRD urban agglomerations. The symbol and definition of the variables involved in this study are summarized in Table 1.

**Table 1:** Variable definitions.

Type	Name	Symbol	Definition
*Outcome variable*	Enterprise innovation	*RDoutput*	Ln (number of patent applications +1)
*Running variable*	Score	*dist*	The shortest distance from the location of enterprises to the boundary of urban agglomerations
*Control variable*	Operating status	*sale*	Ln (operation income)
	Profitability	*profit*	Ln (net profits)
	Owner’s equity	*equity*	Ln (total owners’ equity)

In this study, the quantity of patent applications (*RDoutput*) is chosen to measure the innovation of enterprises for two reasons: first, the quantity of patent applications is not easily interfered by external factors, such as bureaucratic factors, patent maintenance fees; second, the patent application data is easy to obtain, and can be used as a stable and objective standard to effectively measure the innovation [62].

Running variable is set as the shortest distance (*dist*) from the longitude and latitude coordinates of the geographical location of the enterprise to the boundary of urban agglomerations. To obtain the shortest distance for each enterprise, we adopt the following steps. First, the shape of YRD and PRD urban agglomerations defined by government is projected to the ArcGIS software (version 10.5), to obtain the boundary information about urban agglomeration. Second, the geocoding technique, which is the process of converting addresses of enterprises into a coordinate system (typically latitude and longitude), is used to help us to know the precise location of enterprises. Third, we use a buffer to identify which enterprises are within 100km from the border on either side. Fourth, we calculate the shortest distance from each enterprise location to the boundary of urban agglomerations by ArcGIS software, and develop a running variable (score) that reflects the two-dimensional geographic space.

Control variables mainly cover enterprise operating status, enterprise profitability, and owner’s equity of enterprises. Operating status usually affect the innovation behavior and willingness of enterprises. Considering that operating income can reflect the change of short-term demand, the operating income processed by logarithm (*sale*) is used in this study to measure the operating status of enterprises. Enterprise innovation needs a large amount of capital investment. Generally, the higher the profit rate, the higher the level of innovation [63]. Thus, we use the logarithm of net profit (*profit*) to measure the profitability of enterprises. Owners’ equity is the portion of an enterprise’s assets that belongs to the owner after the deduction of creditors’ equity, which can reflect the case of owner investment capital preservation and appreciation. We use the owners’ capital input, other comprehensive income and retained income to measure the owners’ equity (*equity*) of enterprises.

**Fig 2:**
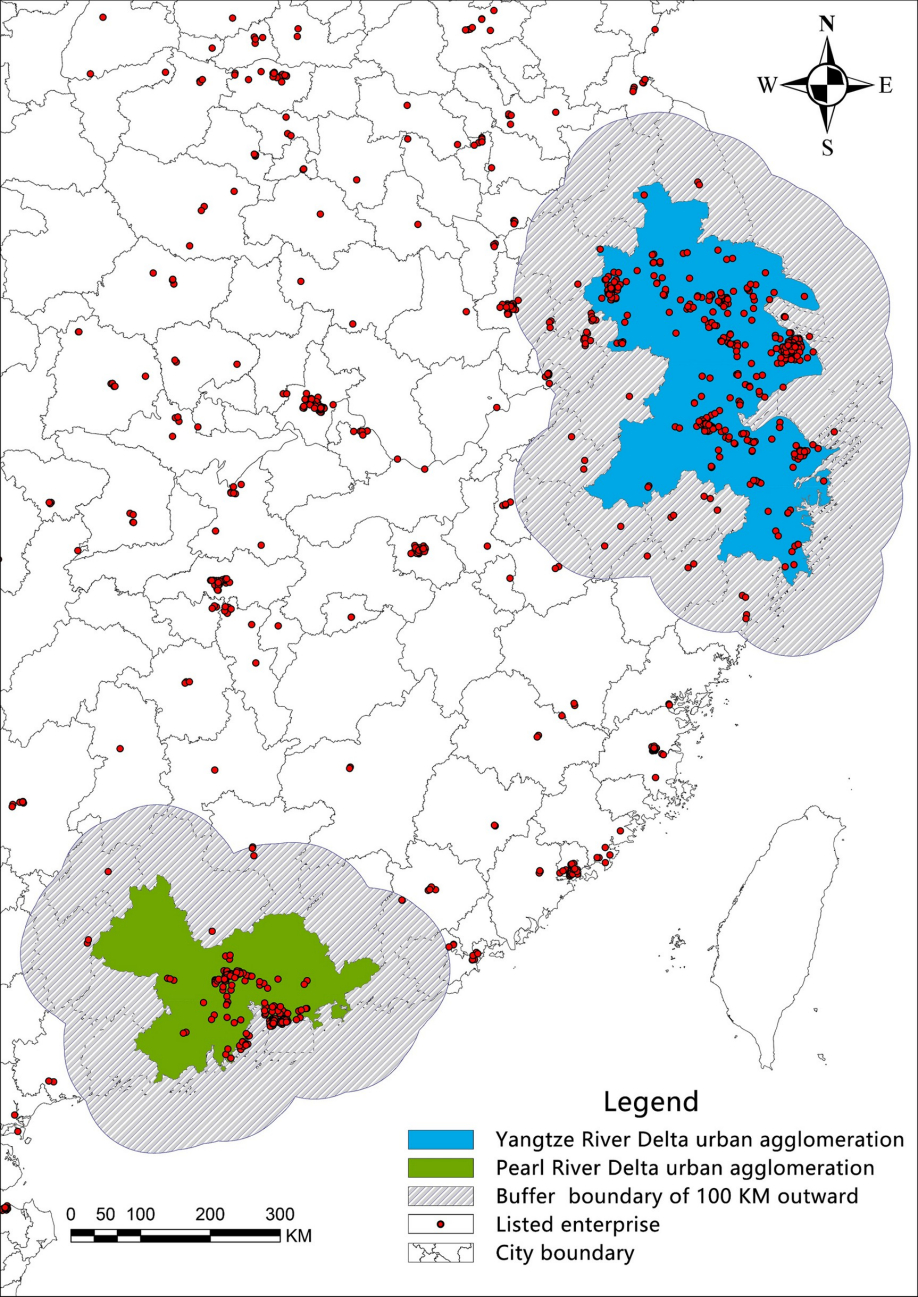
Boundary and buffer of urban agglomerations.

It is worth noting that there are differences in the distribution of enterprise samples on both sides of the boundary of urban agglomerations, specifically more samples within urban agglomeration and less samples outside urban agglomeration. Therefore, we randomly select 288 listed enterprises distributed on both sides of the boundary of urban agglomeration and less than 100 kilometers away from the boundary. After random sampling, there are 149 listed enterprises (109 enterprises in YRD urban agglomeration, 40 enterprises in PRD urban agglomeration) in the treatment group and 139 listed enterprises (121 enterprises in YRD urban agglomeration, 18 enterprises in PRD urban agglomeration) in the control group. Details are shown in Fig 2.

Table 2 lists descriptive statistics for the main variables. It is worthwhile to note that according to *dist*_−_, there are 139 samples of listed enterprises outside the demarcated scope of YRD and PRD urban agglomerations, and the number of enterprises within YRD and PRD urban agglomerations is 149 according to *dist*_+_.

**Table 2:** Descriptive statistics.

Name	Mean	Median	Variance	Minimum	Maximum	Observation
*RDoutput*	3.56	3.95	2.21	0	10.68	288
*dist*	-13.64	0.90	35.18	-96.07	35.63	288
*dist*_−_	-43.88	-52.20	25.86	-96.07	-1.90	139
*dist*_+_	14.58	13.15	10.77	0.09	35.63	149
*sale*	22.13	22.06	1.279	19.09	26.47	287
*profit*	21.76	21.71	1.115	19.41	25.49	287
*equity*	19.34	19.20	1.37	15.45	23.85	269

### 4.2 Preliminary exploration

**Fig 3:**
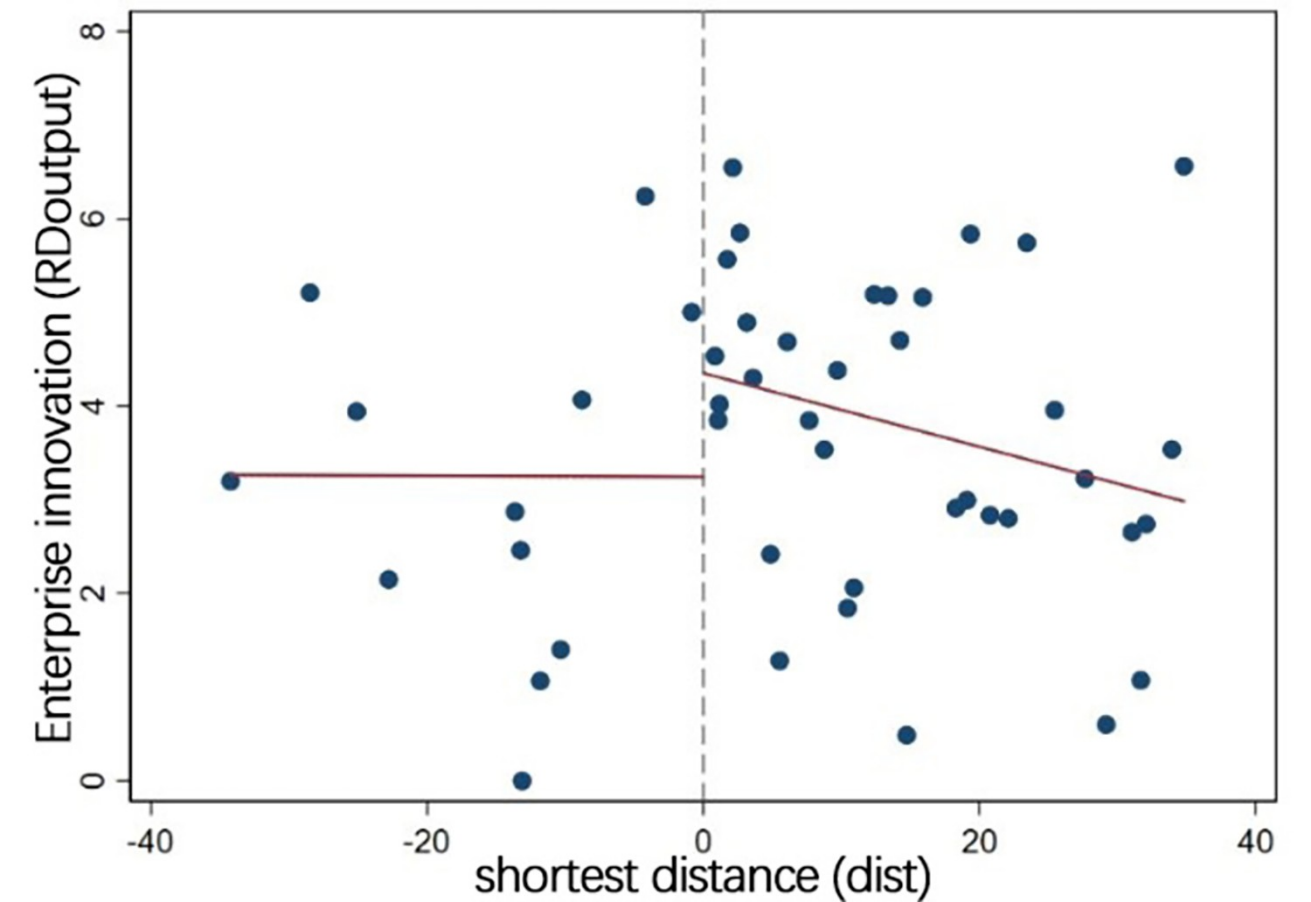
Changes of enterprise innovation at the cutoff.

GRDD can be tested as a randomized experiment. Therefore, before in-depth analysis, we should first observe the breakpoint caused by the policy of the establishment of YRD and PRD urban agglomerations, to verify whether the outcome variable will show systematic changes due to the urban agglomeration setup. Fig 3 shows the relationship between the outcome variable (*RDoutput*) and the running variable (*dist*). It can be seen that enterprise innovation has an obvious jump at the cutoff. This indicates that the innovation level of enterprises in treated group near the boundary of urban agglomerations is significantly higher than that in control group.

**Fig 4:**
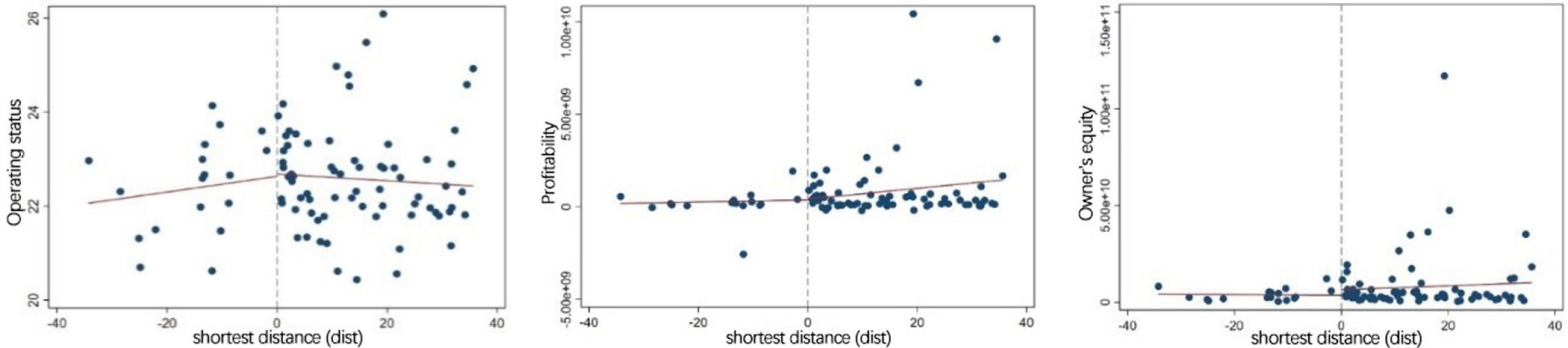
Changes of covariates at the cutoff. a (Operating status). b (Profitability). c (Owner’s equity).

Besides, predetermined covariates (control variables) can be treated as outcomes using the similar approach outlined above to verify whether the change of enterprise innovation is related to the characteristics of enterprises. We would hope to find that there is no obvious jump for operating status (*sale*) and other covariates (*profit*,*equity*) at cutoff (boundary line). Fig 4 shows the changes of covariates. The scatter points are the average value of covariates, and the straight line is the regression fitting value of all the scatter points on both sides of the cutoff. It is shown that all control variables have continuity at the cutoff, and the difference in enterprise innovation at cutoff is not due to the influence of control variables.

## 5 Empirical discussion

### 5.1 Impact of urban agglomeration construction on enterprise innovation

Considering the operating status, profitability, and owners’ equity of enterprises shown in Fig 4 meet the continuity assumption, we introduce the abovementioned control variables into the model estimation. By this way, the actual impact of urban agglomeration establishment on enterprise innovation can be more accurately obtained, and the estimated values is closer to the real values. The specific estimated results are shown in Table 3.

**Table 3:** Estimated results of GRDD.

Outcome	Enterprise innovation			
	(1)	(2)	(3)	(4)
**ATE**	0.831*	0.753*	0.913**	0.921*
	(0.4716)	(0.4294)	(0.4559)	(0.4794)
**Kernel function**	Tri	Tri	Epa	Uni
**Bandwidth (km)**	50	50	50	50
**Control variable**	no	Yes	yes	yes
**Observation**	211	197	197	197

Note

***, **, and * represent the significance levels at 1%, 5%, and 10%, respectively; robust standard error is presented in parentheses; Kernel function “Tri” stands for Triangular; “Epa” stands for Epanechnikov; “Uni” stands for Uniform.

Column (1) of Table 3 shows the estimated results without control variables, columns (2) to (4) are the estimates for introducing control variables and using different kernel functions. It is shown that at the statistical level of 5% or 10%, the average treatment effect (ATE) of urban agglomeration construction on enterprise innovation is significantly positive, and the estimated values of this jump remains between 0.753 and 0.921. No matter whether to introduce control variable or change the form of kernel function, the estimated results are basically consistent, which indirectly confirms the robustness of the study. On the whole, this finding indicates that the urban agglomeration construction can significantly improve the innovation of enterprises within the urban agglomeration. The reason behind this may be that the construction of urban agglomeration is conducive to the formation of agglomeration effect among enterprises. Agglomeration effect can be divided into specialized economic effect and diversified economic effect. The former refers to the gain of enterprises in the same industry due to spatial agglomeration, while the latter refers to the benefit from different industries due to spatial agglomeration. Enterprises in the same industry gather in urban agglomeration, which can bring many benefits to enterprises [64]. First, the agglomeration of enterprises in the same industry can generate knowledge spillover effect [65], which will reduce the learning cost among enterprises and accelerate the speed of knowledge updating [66]. Second, the agglomeration of enterprises in the same industry is also conducive to realizing economies of scale. The convenience of raw material supply and customer group search will be greatly increased, thus reducing the production cost and sales cost of unit product [67]. Third, enterprises in the same industry have a high similarity in the demand for professional personnel. The agglomeration of enterprises expands the development space of professionals, which is conducive to attracting a large number of talents, thus making it easier for enterprises to gather all kinds of technical personnel needed [68]. Fourth, the similarity in the use of factories and equipment among enterprises in the same industry makes it easier to realize the special assets invested by enterprises, which will greatly increase the financial flexibility of enterprises and better meet the capital needs of enterprise innovation [69]. It is thus clear that the specialized economic effects produced by urban agglomeration provide superior conditions for enterprise innovation [70]. The agglomeration of enterprises in different industries in the same urban agglomeration will also generate increasing benefits, even more than that in the same industry [71], namely Jacobs external economy. First, infrastructure can be shared among diversified industries, for example, more banking institutions make corporate financing more convenient and cheaper. Second, the agglomeration of enterprises from different industries in the same urban agglomeration will produce spillover effects of knowledge and technology, and the knowledge spillover or networks could improve the innovation performance of enterprises [72], even lead to the breakthrough innovation [73].

### 5.2 Robustness check

To verify the validity of the previous estimates and prove that the GRDD results do not depend on the special settings of the model, we will conduct the following robustness tests: (1) test for precise control of running variable by enterprises; (2) test for the continuity of control variables; (3) test for bandwidth sensitivity; (4) placebo test with pseudo-boundary; (5) extreme value test.

#### (1) Test for precise control of running variable by enterprises

**Fig 5:**
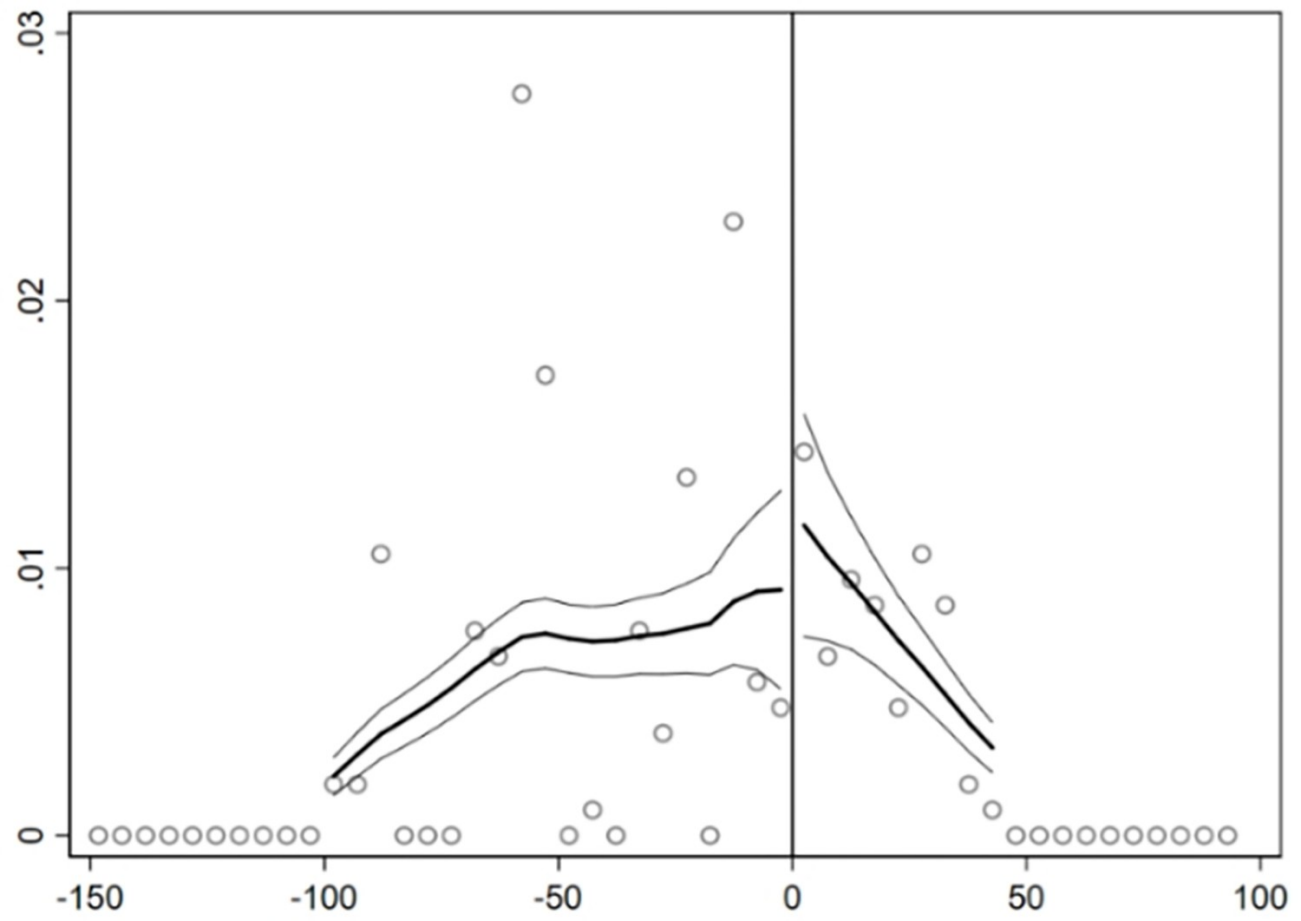
Distribution of running variable.

The premise of GRDD is that the groups on both sides of cutoff (line) have randomness. If sample enterprises can manipulate and even select groups, the estimated results of GRDD will be invalid. To ensure the accuracy and validity of GRDD, it is necessary to test whether the sample enterprise can control the running variable. If the GRDD is valid (there is no manipulation around the cutoff), then there should be no discontinuity observed in the number of observations just above or below the cutoff. We use the nonparametric statistical test provided by McCrary to check whether the distribution of running variable is continuous [74]. Fig 5 depicts the running variable does not jump significantly at the cutoff, and the shortest distance from the enterprise location to the urban agglomeration boundary is continuously distributed. This indicates that the individual enterprise cannot accurately control the running variable, the research design meets the assumptions of GRDD, and the estimated results are valid.

#### (2) Test for the continuity of control variables

To ensure that the urban agglomeration establishment is the cause of the significant change of enterprise innovation, the continuity of the conditional density of control variables near the cutoff should be guaranteed. If the control variable jumps significantly at the cutoff, it means that the jump of enterprise innovation at the cutoff cannot be attributed to the establishment of urban agglomeration. On the basis of Fig 4, we use RDD to further empirically check whether enterprise characteristics change significantly at the cutoff, and to verify the continuity of covariate. Table 4 reports the estimated results of the jump at the cutoff for control variables (covariate). By using three different kernel functions (Triangular, Epanechnikov, and Uniform), we find that the estimated results for all control variables at the cutoff are insignificant. Therefore, there is no empirical evidence that these control variables are discontinuous at the cutoff, which satisfies the validity assumption of GRDD.

**Table 4:** Continuity test of control variables.

	Control variables		
Kernel function	*sale*	*equity*	*profit*
Triangular	0.179	0.225	0.535
	(0.3362)	(0.2376)	(0.3512)
Epanechnikov	0.236	0.331	0.0720
	(0.3249)	(0.2405)	(0.6224)
Uniform	0.315	0.443	-0.468
	(0.3218)	(0.2472)	(0.7253)

Note

***, **, and * represent the significance levels at 1%, 5%, and 10%, respectively; robust standard error is presented in parentheses.

#### (3) Test for bandwidth sensitivity

Since there is a “bias-variance tradeoff”, the choice of bandwidth is fundamental for the analysis and interpretation of GRDD. Table 5 reports the estimated results by using different bandwidths. “BW” represents the initial bandwidth; “BW+1” means adding 1 to the initial bandwidth and “BW+2” means adding 2 to the initial bandwidth, and so on. The test results show that despite the change of bandwidth, the estimated ATEs are significant at the statistical level of 10%, and the difference of estimated values is small (between 0.720 and 0.818). It can be considered that the bandwidth change has no significant impact on the estimated results, and the findings by GRDD shown in Table 3 are robust.

**Table 5:** Sensitivity test to bandwidth choice.

	(1) BW-4	(2) BW-2	(3) BW	(4) BW+2	(5) BW+4
**ATE**	0.720*	0.738*	0.753*	0.765*	0.818*
	(0.4235)	(0.4266)	(0.4294)	(0.4319)	(0.4202)
**Bandwidth selection**	46	48	50	52	54
**Control variable**	Yes	yes	yes	yes	yes
**Observation**	204	206	211	212	220

Note

***, **, and * represent the significance levels at 1%, 5%, and 10%, respectively; robust standard error is presented in parentheses.

#### (4) Placebo test

This falsification test replaces the true boundary by another pseudo-boundary at which the treatment status does not really change, and performs estimation and inference using this fake or placebo cutoff line. The expectation is that no significant treatment effect will occur at placebo cutoff line (pseudo-boundary). If the outcome variable at the pseudo-boundary has a significant jump, it can be considered that some unobserved factors may affect the outcome variable. To this end, we define some pseudo-boundaries, such as “real boundary -1km”, “real boundary +1km”, “real boundary -2km” and “real boundary +2km”. The estimated results based on these four pseudo-boundaries are sorted out in Table 6. It is shown that the ATEs are not statistically significant, and the outcome of interest does not jump discontinuously at the artificial cutoffs considered.

**Table 6:** Placebo test with pseudo-boundary.

	(1)	(2)	(3)	(4)
**ATE**	-0.552	0.326	-1.378	0.065
	(0.5735)	(0.3618)	(0.9121)	(0.3559)
**Pseudo-boundary**	real boundary -1km	real boundary +1km	real boundary -2km	real boundary +2km
**Optimal bandwidth (km)**	8.8	6.3	9.3	5.3
**Kernel function**	Triangular	Triangular	Triangular	Triangular
**Observation**	62	53	63	52

Note

***, **, and * represent the significance levels at 1%, 5%, and 10%, respectively; robust standard error is presented in parentheses.

#### (5) Extreme value test

The sample can be limited to a value of running variable between the upper and lower quartiles, and the extreme value test is carried out by eliminating potential extreme enterprises. GRDD is used to estimate the policy effect with three different kernel functions, and the estimated results are shown in Table 7.

**Table 7:** Extreme value test.

	(1)	(2)	(3)	(4)
**ATE**	1.160**	1.050**	1.201**	1.211**
	(0.5028)	(0.4622)	(0.4901)	(0.5111)
**Kernel function**	Triangular	Triangular	Epanechnikov	Uniform
**Bandwidth (km)**	50	50	50	50
**Control variable**	no	yes	yes	yes
**Observation**	139	127	127	127

Note

***, **, and * represent the significance levels at 1%, 5%, and 10%, respectively; robust standard error is presented in parentheses.

According to results shown in Table 7, the ATEs of the establishment of urban agglomeration on enterprise innovation have some changes compared with the full sample, but the differences are not obvious. The results are not affected by the extreme values, which confirms the reliability of the GRDD estimation in this study.

### 5.3 Influence mechanism of state-level urban agglomeration

Considering that the establishment time of state-level urban agglomerations is different, we follow the practice of Hoynes et al. [75], and adopt Staggered DID to explore the influence mechanisms of state-level urban agglomerations promoting enterprise innovation, specific as follows.

$$RDoutput_{it}=\alpha_{0}+\alpha_{1}\cdot treat_{it}+\alpha_{2}\cdot treat_{it}\times M+X_{it}g+u_{i}+\lambda_{t}+\varepsilon_{it}$$ (8)

where *i* indexes the enterprise, *t* represents the year, *RDoutput* is enterprise innovation. **X** is a set of control variables that change with time (shown in Table 1). *u*_*i*_ is the individual effect, *λ*_*t*_ is the time fixed effect, and *ε*_*it*_ is the random disturbance term. *treat*_*it*_ represents the policy treatment variable. If the enterprise *i* is within the scope of the state-level urban agglomeration in *t* year, this enterprise will be in the treatment period, and the value *treat* of the current year (*t*) and the subsequent period (*t*+1,*t*+2,…) will be 1, otherwise 0. **M** is the mediator variable which stands for financial support channel (*sub*) and regional coordination channel (*coor*), respectively. *sub* is calculated by natural logarithm of government subsidies received by each enterprise plus 1, and the data of government subsidies stem from CSMAR database and annual reports of listed enterprises. As far as the measure of regional coordination effect is concerned, for enterprises out of urban agglomeration, *coor* is measured by the attractiveness score of the city where enterprises are located; for enterprises within urban agglomeration, *coor* is measured by the average score of all node cities in this urban agglomeration. The data of attractiveness score are compiled from the annual “Ranking of Cites’ Business Attractiveness in China” published by Yicai (https://www.yicai.com/).

Column (1) of Table 8 reports the impact of state-level urban agglomeration construction on enterprise innovation using Staggered DID method. The estimation results show that urban agglomeration can significantly promote enterprise innovation, which further verifies the previous findings. According to the results in column (2), the coefficient of cross-term *treat*×*sub* is significantly positive, which proves that urban agglomeration has significant financial support effect on enterprise innovation. The financial support (for example, subsidy) enjoyed by enterprises in urban agglomeration is an important driving force to encourage enterprise innovation. Enterprises in state-level urban agglomerations often have more opportunities to get financial support from the government, which stimulates them to carry out innovation activities [76]. In addition, financial support will indirectly guide the flow of financial resources into urban agglomerations (for example, banks will be more willing to provide low-interest loans to enterprises within urban agglomerations), thus improving innovation by reducing the financing costs of enterprises. Column (3) reports the effect of cross-term *treat*×*coor* on enterprise innovation. The results show that urban agglomeration promotes enterprise innovation through regional coordination channel. The construction of urban agglomeration not only expands the market scale, but also broadens the scope of innovation resources flow, greatly promoting the flow of talents, capital and information in the region [77]. On this basis, the agglomeration of innovation resources and the acceleration of their flow force enterprises in urban agglomerations to improve their R&D modes, thus enhancing their innovation ability.

**Table 8:** Influence mechanism.

	(1)	(2)	(3)
*treat*	1.268***	1.032***	1.041***
	(0.0773)	(0.2909)	(0.2706)
*treat*×*sub*		0.079*** (0.0171)	
*treat*×*coor*			0.763*** (0.1511)
**Control variable**	yes	Yes	yes
**Adj R2**	0.129	0.131	0.132
**Observation** ^**a**^	3288	3288	3288

Note

^**a**^ no sampling process and no control of the distance between sample enterprises and the boundary of urban agglomeration

***, **, and * represent the significance levels at 1%, 5%, and 10%, respectively; robust standard error is presented in parentheses.

## 6 Conclusion

At present, China’s high-quality development needs to be driven by point to area policy, and the establishment of urban agglomeration is undoubtedly in line with this. The YRD is an important intersection of the Belt and Road (B&R) and the Yangtze River Economic Belt; the PRD is one of the most dynamic economic zones in the Asia-Pacific region. Both of them are bases for scientific and technological innovation, important platforms for China to participate in economic globalization, strategic areas for China’s modernization. This study evaluates the impact of YRD and PRD urban agglomeration establishment on enterprise innovation measured by the number of patents using the quasi-natural experimental method GRDD. Unlike the existing literature focusing on regional outcomes, this study explores the impact on individual enterprises based on the data of Chinese listed enterprises. The shortest distances from the enterprise location to the boundary of urban agglomerations are calculated by ArcGIS, and we consider the shortest distance as the running variable. Compared with other methods, the causal inference of GRDD is clear, and the assumptions are easy to test. It is shown that the establishment of YRD and PRD urban agglomerations can significantly improve the enterprise innovation, and this outcome is verified by some robustness tests including bandwidth sensitivity test, placebo test, extreme value test, *etc*. In addition, the influence mechanisms of state-level urban agglomerations promoting enterprise innovation are explored by Staggered DID. It is confirmed that the urban agglomeration construction can promote enterprise innovation through financial support and regional coordination channels.

Overall, the establishment of YRD and PRD urban agglomerations achieves good results in stimulating the innovation of enterprises. It is an important way for China to implement and promote this innovation-driven and place-based strategy to weaken the restrictions of administrative divisions. Although some cities in YRD and PRD are still in an environment with relatively backward resources, the establishment of urban agglomeration can integrate and share the resources within the region, which is conducive to the enterprise innovation and promote the high-quality development. To further strengthen the positive effect of YRD and PRD urban agglomeration on enterprise innovation, relevant government departments should weaken the restrictions of traditional administrative divisions, take urban agglomerations as regional units [78], gather innovative talents in science and technology, optimize the allocation of innovation resources in urban agglomerations and improve infrastructure construction. In addition, the policy implementation environment [79], the thermal comfort environment [80, 81], the infrastructure environment [82, 83] and the innovation environment [84, 85] have important influence on enterprise innovation. Therefore, relevant government departments should build the relatively fair and transparent policy implementation environment, improve the quality of infrastructure, and strengthen Industry-University-Research cooperation, to further promote enterprise innovation. In particular, the relevant departments should pay attention to promoting the steady development of enterprise innovation by building an innovation-driven policy system with urban agglomeration as the carrier. Besides, relevant departments can ameliorate the innovative mode of YRD and PRD urban agglomeration, and take advantage of the “joint development” and “relying” mode of urban agglomeration integration to achieve “introduction, absorption and re-innovation”.

It is unavoidable that some shortcomings remain in this study. First, the measurement of enterprise innovation is rather rough, without considering the differences in patents (for example patent for Invention, patent for Utility Model and patent for Industrial Design). The follow-up research can make a more detailed division of enterprise innovation according to Chinese patent classification standards, so as to reflect the differences in quantity and quality of enterprise innovation. Second, the industries to which the enterprises belong are not refined. In the future, we can further study the influence of YRD and PRD urban agglomerations on innovation activities of enterprises in different industries, and explore the impact of urban agglomeration construction for different industries. Third, the establishment of YRD and PRD urban agglomerations may generate heterogeneous effects on enterprise innovation, it will be better that the different regional policies and the differences of GRDD results can be highlighted in the future study. Fourth, there is a lack of investigation on the dynamic effect of YRD and PRD urban agglomerations. In the future, a variety of causal identification methods can be used to evaluate the actual effect of the “development” and “capacity expansion” of urban agglomeration, and explore the radiation effect of YRD and PRD urban agglomeration on the innovation activities of enterprises outside the boundary of urban agglomerations.
